# Supplementary material for: Binge drinking in young people: protocol for a systematic review of neuropsychological, neurophysiological and neuroimaging studies
Source: BMJ Open. 2018 Jul 30;8(7):e023629. doi: 10.1136/bmjopen-2018-023629 (PMC6067338; doi:10.1136/bmjopen-2018-023629)
Supplement: Author's manuscript [file bmjopen-2018-023629.draft_revisions.pdf]

BMJ Open

BMJ Open is committed to open peer review. As part of this commitment we make the peer review history of every article we publish publicly available.

When an article is published we post the peer reviewers' comments and the authors' responses online. We also post the versions of the paper that were used during peer review. These are the versions that the peer review comments apply to.

The versions of the paper that follow are the versions that were submitted during the peer review process. They are not the versions of record or the final published versions. They should not be cited or distributed as the published version of this manuscript.

BMJ Open is an open access journal and the full, final, typeset and author-corrected version of record of the manuscript is available on our site with no access controls, subscription charges or pay-per-view fees (http://bmjopen.bmj.com).

If you have any questions on BMJ Open's open peer review process please email info.bmjopen@bmj.com

BMJ Open

Binge drinking in young people: Protocol for a systematic review of neuropsychological, neurophysiological and neuroimaging studies

Journal:	*BMJ Open*
Manuscript ID	bmjopen-2018-023629
Article Type:	Protocol
Date Submitted by the Author:	16-Apr-2018
Complete List of Authors:	Lees, Briana; University of New South Wales, NHMRC Centre of Research Excellence in Mental Health and Substance Use, National Drug and Alcohol Research Centre<br>Mewton, Louise; University of New South Wales, NHMRC Centre of Research Excellence in Mental Health and Substance Use, National Drug and Alcohol Research Centre<br>Stapinski, Lexine; University of New South Wales, NHMRC Centre of Research Excellence in Mental Health and Substance Use, National Drug and Alcohol Research Centre<br>Squeglia, Lindsay; Medical University of South Carolina, Department of Psychiatry and Behavioral Sciences<br>Rae, Caroline; University of New South Wales, Neuroscience Research Australia<br>Teesson, Maree; University of New South Wales, NHMRC Centre for Research Excellence in Mental Health and Substance Use, National Drug and Alcohol Research Centre
Keywords:	binge drinking, alcohol use, cognitive development, neurodevelopment, Child & adolescent psychiatry < PSYCHIATRY, young adult

SCHOLARONE™
Manuscripts

Title: Binge drinking in young people: Protocol for a systematic review of neuropsychological, neurophysiological and neuroimaging studies

Authors: Lees, B.[1 2], Mewton, L.[1 2], Stapinski, L.[1 2], Squeglia, L. [3], Rae, C. [4] & Teesson, M. [1 2]

[1] *NHMRC Centre of Research Excellence in Mental Health and Substance Use*
[2] *National Drug and Alcohol Research Centre, University of New South Wales, Sydney, NSW, Australia*
[3] *Department of Psychiatry and Behavioral Sciences, Medical University of South Carolina, Charleston, SC, United States*
[4] *Neuroscience Research Australia, University of New South Wales, Sydney, NSW, Australia*

For peer review only

Keywords: binge drinking, alcohol use, cognitive development, neurodevelopment, adolescence, young adult

Corresponding Author:
Briana Lees, BPsych (Hons)
National Drug and Alcohol Research Centre
University of New South Wales
National Drug and Alcohol Research Centre
22-32 King St
Randwick, 2031
Australia
Phone 61 (2) 8936 1012
Email: b.lees@unsw.edu.au

Word count: 3,463

Abstract

Introduction: Binge drinking is the most common pattern of alcohol use among young people in Western countries. Adolescence and young adulthood is a vulnerable developmental period and binge drinking during this time has a higher potential for neurotoxicity and interference with ongoing neural and cognitive development. The purpose of this systematic review will be to assess and integrate evidence of the impact of binge drinking on cognition, brain structure, and function in youth aged 10 to 24 years. Cross-sectional studies will synthesise the aberrations associated with binge drinking, while longitudinal studies will distinguish the cognitive and neural antecedents from the cognitive and neural effects that are a consequence of binge drinking.

Methods and analysis: A total of five peer-reviewed databases will be systematically searched and the search period will include all studies published prior to 1 April 2018. The search terms will be a combination of MeSH keywords that are based on previous relevant reviews. Study selection will follow the Preferred Reporting Items for Systematic Reviews and Meta-Analyses (PRISMA) guidelines and study quality will be assessed using The Grades of Recommendation, Assessment, Development and Evaluation (GRADE) approach. All studies will be screened against eligibility criteria designed to synthesise studies that examined a young binge drinking sample and used neuropsychological, neurophysiological, or neuroimaging assessment techniques. Studies will be excluded if participants were significantly involved in other substances or if they had been clinically diagnosed with an alcohol use disorder, or any psychiatric, neurological, or pharmacological condition. If available data permits, a meta-analysis will be conducted.

Ethics and dissemination: Formal ethics approval is not required as primary data will not be collected. The results will be disseminated through a peer-reviewed publication, conference presentations and social media.

Trial registration number International Prospective Register for Systematic Reviews (PROSPERO) number: CRD42018086856.

Strengths and limitations of this study

- This systematic review and meta-analysis will be the first to synthesise neuropsychological, neurophysiological, and neuroimaging studies examining the developmental impact of binge drinking on cognition, brain structure, and function in youth.
- This review will report on cross-sectional and longitudinal data to firstly identify the cognitive and neural aberrations associated with binge drinking, and secondly to distinguish the antecedents of binge drinking from the effects caused by binge drinking.
- Identified cognitive and neural precursors and consequences of binge drinking will be informative for prevention, early intervention, and treatment efforts.

For peer review only

Introduction

Alcohol misuse amongst young people is widely recognised as a global health priority [1] and has raised concern about the neurotoxic effects of alcohol use on a large scale [2]. Binge drinking is a pattern of alcohol use that brings blood alcohol concentration levels to 0.08 g/dL which typically occurs after the consumption of four or more alcoholic drinks per drinking occasion (i.e., at the same time or within a couple of hours of each other) for females and five or more drinks per occasion for males [3, 4]. This episodic pattern of drinking, where an individual drinks less frequently but in larger amounts, is most common among adolescents in Western countries [5-7]. For instance, approximately 10-16% (United States; US), 23% (United Kingdom) and 15% (Australia) of 15-17 year olds report binge drinking in the previous month [8-10]. The prevalence of binge drinking sharply increases from adolescence to young adulthood, with 40-50% of young adults reporting binge drinking at least monthly [6, 9, 11-13] . Extreme binge drinking (defined as 10 or more drinks per occasion) is also common, with 29% of young adults in Australia and 16% of US adolescents engaging in this behaviour [9, 14, 15]. This is concerning because early alcohol use and binge drinking is associated with a myriad of short- and long-term negative consequences including blackouts, hangovers and alcohol poisoning [16, 17], alcohol and drug use disorders [18-20], other mental health problems [21], risky sexual behaviours [22, 23], injuries [24, 25] and increased risk of being a victim of assault or accidental death [26, 27].

Studies consistently indicate that alcohol use and misuse during adolescence (10-19 years) and young adulthood (20-24 years) has a higher potential for neurotoxicity and interference with ongoing neural and cognitive development than during later adulthood [16, 25, 28-36]. This is because adolescence and young adulthood is a vulnerable developmental period characterised by significant neural changes. Although brain size is thought to stabilise around the age of five years [37], important morphometric restructuring and functional neuromaturation continues to occur during adolescence and young adulthood with substantial myelinisation, synaptic refinement, and changes in grey and white matter volume until the age of 25 years [38]. The reward and mesolimbic systems mature during mid-adolescence, prior to the development of prefrontal and cognitive control regions which continue to develop into late adolescence [39-42]. This has a two-fold effect, firstly this hypersensitivity to reward during adolescence results in an increased propensity to engage in risky and sensation-seeking activities, including drug and alcohol use [43]. Secondly, risky drinking during prefrontal brain development may interfere with neuromaturation and translate to ongoing neural aberrations and top-down cognitive processing deficits, reducing youth's ability to enable self-control and resist temptations (inhibition); to see reason, problem solve, and consider alternatives (working memory);

For peer review only

and to plan and change perspective (cognitive flexibility) [44]. Collectively, these changes in cognitive processing may lead to increased motivation to consume alcohol and a decreased ability to regulate this motivation and drinking behaviour. As many of these developmental changes occur in brain regions that appear to be particularly sensitive to alcohol [45, 46], it is critical that research examines the associated negative consequences of risky episodic drinking during a vulnerable developmental period as the neural insults may have ongoing cognitive and behavioural impacts.

The growing concern about alcohol use amongst young people has led to a significant increase in the number of studies using neuropsychological, neurophysiological, and neuroimaging techniques to determine the effects on brain and cognitive development. Over the past decade there has also been a rise in the number of longitudinal designs that assess young people before they initiate alcohol use and continue to assess them over time as a portion begin to initiate use. These prospective longitudinal studies have made it possible to disentangle the antecedents and consequences of alcohol use in young people. A recent review of longitudinal studies that concentrated on alcohol initiation in adolescence found that reduced grey matter volume (frontal), less white matter volume (cerebellar, nucleus accumbens, anterior cingulate), poor white matter integrity (fronto-limbic), decreased activation during inhibition and working memory tasks, and increased reward response (frontal) were antecedents of alcohol use initiation in adolescence [47]. Accelerated decreases in grey matter (frontal, temporal), attenuated white matter development (pons, corpus callosum), poor white mater integrity, and increased brain activation during inhibition and working memory tasks were reported consequences following alcohol use initiation. In terms of cognitive domains, poorer inhibitory functioning and working memory were antecedents of alcohol initiation in adolescence while poorer verbal learning and memory, visuospatial functioning, psychomotor speed and working memory were reported effects following alcohol use initiation in adolescence. By distinguishing the antecedents from the consequences of alcohol use initiation, this review provides researchers with specific neural and cognitive domains to target in prevention and treatment efforts.

Considering binge drinking is the dominant pattern of use among young people, it is important to understand the neural and cognitive impact this pattern of drinking has on the developing brain. Several narrative reviews (summarising neuropsychological, neurophysiological, and neuroimaging studies; see [7, 17, 28, 45, 48, 49]) and one systematic review from 2014 (including only neuroimaging studies [50]) have summarised the recent binge drinking literature. The systematic review concluded

For peer review only - http://bmjopen.bmj.com/site/about/guidelines.xhtml

that there were a number of structural changes associated with binge drinking, including smaller grey and white matter volume compared to non-binge drinkers [29, 51] and lower white matter integrity across more than 18 white matter regions [52, 53]. Functional differences reported in binge drinkers included less activation during a spatial working memory task [54] and abnormal activation during verbal encoding [55] and decision-making tasks [56]. In terms of neuropsychological studies, narrative reviews have concluded that binge drinking is associated with several cognitive deficits, including impairments in verbal, non-verbal and spatial working memory, as well as attention and executive function [33-35, 57]. A review of neurophysiological studies found that young binge drinkers displayed latency differences in several ERP components, including P1, N1, P3, P3b and P450, in response to a number of cognitive functioning tasks [28]. The early positive and negative voltage deflections (P1, N1) reflect initial sensory differences between binge drinkers and non-binge drinkers, while the later components (P3, P3b, P450) reflect differences in the way participants processed the cognitive tasks. Overall, there is a growing evidence base that consistently demonstrates neural and cognitive aberrations associated with binge drinking. More recently, there has been an increase in the number of prospective longitudinal studies examining the effect of binge drinking among young people. Integrating these new findings is essential to understanding whether the neural and cognitive precursors and consequences of binge drinking are similar or divergent to the domains related to alcohol initiation in adolescence.

The aim of this systematic review is to therefore provide an update on the expanding literature and synthesise the neuropsychological, neurophysiological, and neuroimaging literature on binge drinking and neurodevelopment. This review will also address limitations identified in the previous systematic review. The authors of the 2014 systematic review limited their search to one peer-reviewed database, included adolescents aged 10-19 years and included concurrent substance use. Searching a broader range of peer-reviewed databases may identify studies which were potentially missed in the previous review. Expanding the age range to include young people aged 10-24 years aligns with evidence that neuromaturation continues into the mid-20s [38], as well as the World Health Organisation's definition of young people [58]. To examine the specific effect of binge drinking on brain development and functioning, studies should exclude individuals with concurrent regular use of drugs other than alcohol [45, 47] as well as exclude samples that characterised drinking based on non-binge, heavy-drinking or diagnostic criteria, including alcohol abuse, dependence and alcohol use disorder [49]. Alcohol use disorder is characterised by continued alcohol use despite clinically significant social and physiological consequences. Therefore, the type, extent and magnitude of the neural and cognitive aberrations associated with alcohol use disorder are likely to differ from those

For peer review only

associated with adolescent binge drinking. Additionally, no review has systematically integrated neuropsychological, neurophysiological, and neuroimaging data. Integrating neuropsychological and neurophysiological studies with neuroimaging research is crucial because cognitive processes make an important contribution to excessive alcohol consumption [57] and assessing this data conjointly will provide a broader understanding of the impact binge drinking has on brain development and behaviour. Finally, previous reviews have not critically appraised the within-study risk of bias or overall quality of the body of literature.

This review will involve conducting a systematic literature search of cross-sectional and longitudinal studies to assess and integrate the evidence regarding the impact of binge drinking on cognition, brain structure, and function, utilising neuropsychological, neurophysiological (electroencephalography; EEG, event-related potential; ERP) and neuroimaging (magnetic resonance imaging; MRI, functional MRI; fMRI, diffusion tensor images; DTI) studies. If available data permits, a meta-analysis will be conducted to determine the overall effects of binge drinking on the outcomes of interest. By including cross-sectional studies, we aim to synthesise the cognitive and neural aberrations associated with binge drinking in young people. On the other hand, longitudinal studies that track individuals over time will distinguish cognitive and neural antecedents that predict later binge drinking from the cognitive and neural effects that are a consequence of binge drinking during adolescence and young adulthood (see Figure 1 for logic model). Due to the limited number of published longitudinal studies at the time of the previous systematic review, this systematic review will be the first to infer causality. The predisposing and consequential factors may not be mutually exclusive and some of the vulnerability factors that predict binge drinking behaviour may also be further impacted by the initiation and continuation of binge drinking. Importantly, identified precursors of binge drinking will be informative for prevention and early intervention efforts. Meanwhile, by identifying consequences of binge drinking, treatment efforts will be able to pursue targeted cognitive and physiological training to determine whether these neural insults have ongoing cognitive and behavioural impacts or whether they can recover following a decrease in alcohol use.

[Insert Figure 1]

For peer review only

Objectives

1. To assess and integrate evidence of the impact of binge drinking on cognitive, structural, and functional development in people aged 10-24 years, compared to healthy controls who do not meet the criteria for binge drinking.
2. To synthesise the cognitive and neural aberrations associated with binge drinking by utilising cross-sectional data.
3. To identify the cognitive, structural, and functional features that predispose youth to binge drinking, and separate this from the cognitive, structural and functional features that are a consequences of binge drinking.
4. To examine the within-study risk of bias and assess the quality of the body of evidence examining the relationship between binge drinking and cognitive, structural, and functional deficits in adolescents and young adults.

Methods

This protocol follows the Preferred Reporting Items for Systematic Reviews and Meta-Analysis Protocols (PRISMA-P) statement [59], found in the Supplement File (1). This protocol has been registered with the PROSPERO International Prospective Register of Systematic Reviews of the University of York (registration number: CRD42018086856).

Search Strategy

Relevant literature from PubMed, EMBASE, Medline, PsycINFO and ProQuest will be systematically searched using a comprehensive search strategy which was developed using medical subject headings (MeSH). The Ovid Medline search strategy is provided in the Supplement File (2), which will be replicated for the other electronic databases. This strategy will search through all relevant literature published prior to 1 April 2018. A snowballing technique will be employed where the reference list of identified articles will also be screened for suitable studies.

Search terms will be used to identify neuropsychological, neurophysiological, and neuroimaging studies assessing the impact of binge drinking on neurodevelopment and neuropsychological task performance in adolescents and young adults. The search terms will be based on previous reviews examining the association between substance use, cognition, brain structure, and function [16, 50, 60, 61]. Search terms will be combinations of MeSH keywords describing the participants (adolescent,

For peer review only - http://bmjopen.bmj.com/site/about/guidelines.xhtml

teenager, youth, emerging adult, young adult), the exposure variable (alcohol, binge drinking, ethanol), and the assessment methods measuring the outcomes of interest (neuroimaging, brain imaging, MRI, fMRI, DTI, neurophysiological, EEG, ERP, neuropsychological, cognitive, verbal working memory tests, episodic memory tests, visuospatial working memory tests, verbal fluency tests, executive function tests, digit symbol substitution tests, reaction time, attention). Two reviewers will be involved in independently screening articles, extracting data and assessing the methodological quality.

Eligibility Criteria

Eligibility criteria for this review are defined using PICOS (population, intervention/exposure, comparator, outcome, study characteristics). Table 1 provides an overview of the selection criteria.

[Insert Table 1]

Population

Study samples will be limited by age to human adolescents and young adults ranging from 10 to 24 years at first assessment, which is consistent with the World Health Organisation's definition of young people [58]. Studies that include both a sample of young people and an adult sample (>24 years) will be included if the majority of participants are aged 10 to 24 years or if a separate analysis for participants within the age range of this review was provided. A minimum of 12 participants per group (binge, comparator) must be included within the study analysis, consistent with previous reviews in this area [50]. Finally, studies must be available in the English language to be included in this review.

Exposure

Studies must include a binge drinking sample, where binge drinking is defined as four or more drinks per occasion for females or five or more drinks per occasion for males [3, 4]. Consistent with previous reviews in this area, studies will not be included in this review if samples were based on diagnostic criteria (e.g., alcohol abuse or alcohol use disorder) [49], or if majority of the participants were significantly involved with substances other than alcohol (i.e., >5 cannabis use per month, >25 lifetime other drug use occasions [54, 62, 63]). Studies that include participants who smoke tobacco will be included. Participant disclosure of other substance use or a urine sample identifying other substance use will be sufficient to exclude these studies. Studies that included participants who had

For peer review only

other clinically diagnosed psychiatric, neurological, or pharmacological conditions will also be excluded from this review to ensure that outcomes are specific to binge drinking.

Comparator

Inclusion of a control group is required for studies to be included in this review. Studies must compare participants who meet the criteria for binge drinking with healthy controls who do not drink alcohol or who drink low levels of alcohol but do not meet the criteria for binge drinking.

Outcomes

Studies must report empirical data where the primary outcomes of interest are global and regional volume (structural images), global and regional activity (functional images; cerebral blood flow, regional cerebral blood flow, or blood oxygen level dependent signal), white matter integrity (DTI), brain electrical activity (EEG, ERP responses), and cognitive task performance in neuropsychological and fMRI studies. Global measures include grey matter, white matter, cerebral spinal fluid, and total intracranial volume differences between the active and control group. Regional measures include white matter and grey matter (frontal lobe, parietal lobe, temporal lobe, occipital lobe, basal ganglia and cerebellum) differences between the active and control group. For neuroimaging and neurophysiological studies, detailed results of significant findings will be reported. For neuropsychological studies, significant differences in cognitive task performance between the active and control group will be reported.

Study characteristics

Peer-reviewed cross-sectional and longitudinal neuropsychological, neurophysiological, and neuroimaging studies that provide original data and were published before 1 April 2018 will be included. Reviews and information in books or letters will not be included. Any publication that reported data using two or more techniques from the same subject (e.g. structural MRI and functional MRI) will be considered separately in the review.

Selection procedure

Two researchers will be involved in the review and selection procedure. Reviewer one (BL) will screen all titles and abstracts from the peer-reviewed databases to determine eligibility for inclusion in the review. Reviewer two (LM) will independently screen a random selection of 25% of abstracts

For peer review only

to ensure accuracy in the study selection. Cohen's kappa will be calculated to assess the inter-rater agreement between the two reviewers. To ensure a strong level of agreement, a Cohen's kappa of at least 0.8 is required (64). Full-text versions of the potentially eligible studies will be assessed by both reviewers to further determine eligibility for inclusion. Again, Cohen's kappa will be calculated at the full-text screening stage. Consultation between reviewers will be held at the time of abstract screening and full-text assessment to reconcile any differences of opinion. If consensus cannot be reached, a third member of the research team (LS) will review the eligibility of the study.

Data extraction

All citations will be imported into Covidence (65) and Endnote (66). Endnote will be used to store and manage all review data. Covidence will be used to screen titles, abstracts and full texts. Reviewer one will extract data using a data extraction spreadsheet in Excel. Study characteristics will be extracted from published papers, with study authors contacted in the event of missing data. The following information will be extracted from the included studies:

1. *Study information:* names of authors, year of publication, primary outcome measurements, statistical approaches;
2. *Participant characteristics:* sample size, sex, age, handedness, other substance use (i.e., tobacco use, cannabis use that is <5 occasions/month);
3. *Alcohol characteristics:* age of onset, frequency of binge drinking, mean quantity of alcohol consumed;
4. *Study characteristics:* imaging modality and analysis, binge drinking sample and control group criteria, cognitive task performed, cognitive and neural domain measured, neurophysiological activity measured, rest/active condition (for functional imaging studies), and exclusion criteria, including absence of neurological, psychiatric, or pharmacological conditions, alcohol use disorder or significant involvement with substances other than alcohol; and
5. *Results:* results of outcomes of interest for this review.

Data analysis and quality assessment

A table summarising the results will be produced, including information about imaging modality and analysis or neuropsychological tests, sample information, alcohol characteristics, and the study findings. For longitudinal studies, pre-existing cognitive, structural, and functional features will be

For peer review only

separated from cognitive, structural, and functional features that are evident as a consequence of binge drinking. If available data permits, a meta-analysis will be conducted using Comprehensive Meta-Analysis. Hedges' g will be calculated to determine the binge drinking between-group standardised mean effect size from outcomes of interest (global and regional volume, white matter integrity, neural activity, and cognitive performance). A random effects model will be adopted as wide variations in participant characteristics and methodological factors are expected between the studies.

In the case of insufficient homogenous data, a narrative synthesis of the main results extracted from the studies will be completed. The studies will be classified according to the study type (i.e., neuropsychological task, neurophysiological measurement, structural imaging, functional imaging) and a summary of differences identified in the binge drinking sample compared to the control group will be reported in text.

Following data extraction, the quality of each study will be critically appraised using The Grades of Recommendation, Assessment, Development, and Evaluation (GRADE) approach [(67)]. The GRADE system entails an assessment of the quality of a body of evidence for each individual outcome. The GRADE approach defines the quality of the body evidence as the extent to which one can be confident that an estimate of effect or association is close to the quantity of specific interest. This involves considerations of within-study risk of bias (methodological quality of design), directness of evidence, heterogeneity of results, precision of results, and the probability of publication bias. Reviewer one will critically appraise all included studies using the GRADE system. Reviewer two will assess the quality of a random selection of 25% of studies to ensure scoring accuracy. Consultation between reviewers will be held to reconcile any differences of opinion.

Dissemination

The systematic review will be published in a peer-reviewed journal, presented at conferences and will be shared on social media platforms.

Conclusion

This paper summarises the protocol for a systematic review of neuropsychological, neurophysiological, and neuroimaging studies conducted in youth who binge drink. The purpose of this review is to assess and integrate the evidence of the developmental impact of binge drinking on

For peer review only

cognition, brain structure, and function. Cross-sectional studies will be included in order to synthesise the cognitive and neural aberrations associated with binge drinking in young people. Longitudinal data will be sought to distinguish cognitive and neural antecedents of binge drinking from the cognitive and neural effects that are a consequence of binge drinking during adolescence and young adulthood. This review will be the first to synthesise neuropsychological, neurophysiological, and neuroimaging evidence in a systematic way, to include a meta-analysis of the findings, and the first to assess the quality of the body of neuropsychological, neurophysiological and neuroimaging studies. This review aims to provide researchers, policy makers and program developers with identified antecedents and consequences of binge drinking that will be informative for prevention, early intervention and treatment efforts.

For peer review only

References

1. World Health Organization. Global status report on alcohol and health. Geneva; 2014.
2. Patrick ME, Terry-McElrath YM, Kloska DD, Schulenberg JE. High-intensity drinking among young adults in the United States: Prevalence, frequency, and developmental change. Alcoholism, clinical and experimental research. 2016;40:1905-1912.
3. National Institute on Alcohol Abuse and Alcoholism (NIAAA). Drinking levels defined. 2018; https://www.niaaa.nih.gov/alcohol-health/overview-alcohol-consumption/moderate-binge-drinking (Accessed 12 April 2018).
4. Substance Abuse and Mental Health Services Administration (SAMHSA). Binge drinking: Terminology and patterns of use. 2016; https://www.samhsa.gov/capt/tools-learning-resources/binge-drinking-terminology-patterns (Accessed 12 April 2018).
5. Substance Abuse and Mental Health Services Administration (SAMHSA). Results from the 2008 national survey on drug use and health: Summary of national findings. Rockville, MD: Office of Applied Studies; 2009.
6. Archie S, Zangeneh Kazemi A, Akhtar-Danesh N. Concurrent binge drinking and depression among Canadian youth: Prevalence, patterns, and suicidality. Alcohol. 2012;46:165-72.
7. Hermens DF, Lagopoulos J, Tobias-Webb J, De Regt T, Dore G, Juckes L, et al. Pathways to alcohol-induced brain impairment in young people: A review. Cortex. 2013;49:3-17.
8. Windle M. Drinking over the lifespan: Focus on early adolescents and youth. Alcohol Research: Current Reviews. 2016;38:95-101.
9. Australian Institute of Health and Welfare (AIHW). National drug strategy household survey 2016: Detailed findings. Canberra, ACT: AIHW; 2017.
10. Johnston LD, O'Malley PM, Miech RA, Bachman JG, Schulenberg JE. Monitoring the Future national survey results on drug use, 1975-2017: Overview, key findings on adolescent drug use. Ann Arbor: Institute for Social Research, The University of Michigan; 2017.
11. Substance Abuse and Mental Health Services Adminstration (SAMHSA). Results from the 2010 national survey on drug use and health: Summary of national findings. Rockville, MD: Office of Applied Studies; 2011.
12. Australian Institute of Health and Welfare (AIHW). National drug strategy household survey detailed report 2013. Canberra, ACT: AIHW; 2014.
13. Schuckit MA, Smith TL, Heron J, Hickman M, Macleod J, Munafo MR, et al. Latent trajectory classes for alcohol-related blackouts from age 15 to 19 in ALSPAC. Alcoholism Clinical & Experimental Research. 2015;39:108-116.
14. Eaton DK, Kann L, Kinchen S, Shanklin S, Flint KH, Hawkins J, et al. Youth risk behaviour surveillance - United States. Morbidity and Mortality Weekly Report Surveillance Summaries (Washington, DC: 2002). 2012;61:1-162.
15. Patrick ME, Schulenberg JE, Martz ME, Maggs JL, O'Malley PM, Johnston LD. Extreme binge drinking among 12th-grade students in the United States: Prevalence and predictors. JAMA Pediatrics. 2013;167:1019-1025.
16. Zeigler DW, Wang CC, Yoast RA, Dickinson BD, McCaffree MA, Robinowitz CB, et al. The neurocognitive effects of alcohol on adolescents and college students. Preventive Medicine. 2005;40:23-32.
17. Hermens DF, Lagopoulos J. Binge drinking and the young brain: A mini review of the neurobiological underpinnings of alcohol-induced blackout. Frontiers in Psychology. 2018;9:12.
18. Chassin L, Hussong A, Barrera MJ, Molina BSG, Trim R, Ritter J. Adolescent substance use. In: Lerner RM, Steinberg L, editors. Handbook of Adolescent Psychology (2nd ed). Hoboken, NJ, USA: John Wiley & Sons Inc.; 2004. p. 665-96.

For peer review only

19. Grant BF, Dawson DA. Age at onset of alcohol use and its association with DSM-IV alcohol abuse and dependence: Results from the National Longitudinal Alcohol Epidemiologic Survey. Journal of Substance Abuse. 1997;9:103-110.
20. Newton-Howes G, Boden JM. Relation between age of first drinking and mental health and alcohol and drug disorders in adulthood: Evidence from a 35-year cohort study. Addiction. 2016;111:637-644.
21. Welsh JW, Knight JR, Hou SS, Malowney M, Schram P, Sherritt L, et al. Association between substance use diagnoses and psychiatric disorders in an adolescent and young adult clinic-based population. Journal of Adolescent Health. 2017;60:648-52.
22. Halpern-Felsher BL, Millstein SG, Ellen JM. Relationship of alcohol use and risky sexual behaviour: A review and analysis of findings. Journal of Adolescent Health. 1996;19:331-336.
23. Tapert SF, Aarons GA, Sedlar GR, Brown SA. Adolescent substance use and sexual risk-taking behaviour. Journal of Adolescent Health. 2001;28:181-189.
24. Porter RS. Alcohol and injury in adolescents. Pediatric Emergency Care. 2000;16:316-320.
25. Spear LP. Adolescents and alcohol: Acute sensitivities, enhanced intake, and later consequences. Neurotoxicology and Teratology. 2014;41:51-59.
26. Bonomo YA, Bowes G, Coffey C, Carlin JB, Patton GC. Teenage drinking and the onset of alcohol dependence: A cohort study over seven years. Addiction. 2004;99:1520-8.
27. Mundt MP, Zakletskaia LI, Brown DD, Fleming MF. Alcohol-induced memory blackouts as an indicator of injury risk among college drinkers. Injury Prevention. 2012;18:44-49.
28. Jacobus J, Tapert SF. Neurotoxic effects of alcohol in adolescence. Annual Review of Clinical Psychology. 2013;9:703-21.
29. Lisdahl KM, Gilbart ER, Wright NE, Shollenbarger S. Dare to delay? The impacts of adolescent alcohol and marijuana use onset on cognition, brain structure and function. Frontiers in Psychiatry. 2013;4:53.
30. Peeters M, Vollebergh WA, Wiers RW, Field M. Psychological changes and cognitive impairments in adolescent heavy drinkers. Alcohol and Alcoholism. 2014;49:182-186.
31. Squeglia LM, Jacobus J, Tapert SF. The influence of substance use on adolescent brain development. Clinical EEG and Neuroscience. 2009;40:31-38.
32. Squeglia LM, Jacobus J, Tapert SF. The effect of alcohol use on human adolescent brain structures and systems. Handbook of Clinical Neurology. 2014;125:501-510.
33. Brown SA, Tapert SF, Granholm E, Delis DC. Neurocognitive functioning of adolescents: Effects of protracted alcohol use. Alcoholism Clinical & Experimental Research. 2000;24:164-171.
34. Tapert SF, Brown SA. Neuropsychological correlates of adolescent substance abuse: Four-year outcomes. Journal of the International Neuropsychological Society. 1999;5:481-493.
35. Tapert SF, Granholm E, Leedy NG, Brown SA. Substance use and withdrawal: Neuropsychological functioning over 8 years in youth. Journal of the International Neuropsychological Society. 2002;8:873-883.
36. Nixon K, McClain JA. Adolescence as a critical window for developing an alcohol use disorder: Current findings in neuroscience. Current Opinion in Psychiatry. 2010;23:227-232.
37. Durston S, Hulshoff Pol HE, Casey BJ, Giedd JN, Buitelaar JK, van Engeland H. Anatomical MRI of the developing human brain: What have we learned? Journal of the American Academy of Child and Adolescent Psychiatry. 2001;40:1012-1020.
38. Giedd JN. Structural magnetic resonance imaging of the adolescent brain. Annals of the New York Academy of Sciences. 2004;1021:77-85.
39. Casey BJ, Jones RM, Hare TA. The adolescent brain. Annals of the New York Academy of the Sciences. 2008;1124:111-126.
40. Ernst M, Nelson EE, Jazbec S, McClure EB, Monk CS, Leibenluft E, et al. Amygdala and nucleus accumbens in responses to receipt and omission of gains in adults and adolescents. NeuroImage. 2005;25:1279-1291.

For peer review only

41. Koob GF, Volkow ND. Neurocircuitry of addiction. Neuropsychopharmacology. 2010;35:217-238.
42. Sturman DA, Moghaddam B. Reduced neuronal inhibition and coordination of adolescent prefrontal cortex during motivated behavior. The Journal of Neuroscience. 2011;31:1471-1478.
43. Somerville LH, Jones RM, Casey BJ. A time of change: Behavioral and neural correlates of adolescent sensitivity to appetitive and aversive environmental cues. Brain Cognition. 2010;72:124-133.
44. Diamond A. Executive functions. Annual Review of Psychology. 2013;64:135-168.
45. Spear LP. Effects of adolescent alcohol consumption on the brain and behaviour. Nature Reviews Neuroscience. 2018;19:197-214.
46. Crews FT, Vetreno RP, Broadwater MA, Robinson DL. Adolescent alcohol exposure persistently impacts adult neurobiology and behavior. Pharmacological Reviews. 2016;68:1074-1109.
47. Squeglia LM, Gray KM. Alcohol and drug use and the developing brain. Current Psychiatry Reports. 2016;18:46.
48. Petit G, Maurage P, Kornreich C, Verbanck P, Campanella S. Binge drinking in adolescents: A review of neurophysiological and neuroimaging research. Alcohol and Alcoholism. 2014;49:198-206.
49. Cservenka A, Brumback T. The Burden of Binge and Heavy Drinking on the Brain: Effects on Adolescent and Young Adult Neural Structure and Function. Frontiers in Psychology. 2017;8:1111.
50. Feldstein Ewing SW, Sakhardande A, Blakemore S-J. The effect of alcohol consumption on the adolescent brain: A systematic review of MRI and fMRI studies of alcohol-using youth. NeuroImage: Clinical. 2014;5:420-37.
51. Luciana M, Collins PF, Muetzel RL, Lim KO. Effects of alcohol use initiation on brain structure in typically developing adolescents. American Journal of Drug and Alcohol Abuse. 2013;39:345-355.
52. Jacobus J, McQueeny T, Bava S, Schweinsburg BC, Frank LR, Yang TT, et al. White matter integrity in adolescents with histories of marijuana use and binge drinking. Neurotoxicology Teratology. 2009;31:349-355.
53. McQueeny T, Schweinsburg BC, Schweinsburg AD, Jacobus J, Bava S, Frank LR, et al. Altered white matter integrity in adolescent binge drinkers. Alcoholism, Clinical and Experimental Research. 2009;33:1278-1285.
54. Squeglia LM, Schweinsburg AD, Pulido C, Tapert SF. Adolescent binge drinking linked to abnormal spatial working memory brain activation: Differential gender effects. Alcoholism Clinical & Experimental Research. 2011;35:1831-1841.
55. Schweinsburg AD, McQueeny T, Nagel BJ, Eyler LT, Tapert SF. A preliminary study of functional magnetic resonance imaging response during verbal encoding among adolescent binge drinkers. Alcohol. 2010;44:111-117.
56. Xiao L, Bechara A, Gong Q, Huang X, Li X, Xue G, et al. Abnormal affective decision making revealed in adolescent binge drinkers using a functional magnetic resonance imaging study. Psychology of Addictive Behaviors. 2013;27:443-454.
57. Field M, Schoenmakers T, Wiers RW. Cognitive processes in alcohol binges: A review and research agenda. Current Drug Abuse Reviews. 2008;1:263-279.
58. World Health Organization (WHO). Recognizing adolescence. 2014; http://apps.who.int/adolescent/second-decade/section2/page1/recognizing-adolescence.html (Accessed 13 April 2018).
59. Moher D, Shamseer L, Clarke M, Ghersi D, Liberati A, Petticrew M, et al. Preferred reporting items for systematic review and meta-analysis protocols (PRISMA-P) 2015 statement. Systematic Reviews. 2015;4:1.

For peer review only

60. Batalla A, Bhattacharyya S, Yucel M, Fusar-Poli P, Crippa JA, Nogue S, et al. Structural and functional imaging studies in chronic cannabis users: A systematic review of adolescent and adult findings. PLoS ONE. 2013;8:e55821.
61. Welch KA, Carson A, Lawrie SM. Brain structure in adolescents and young adults with alcohol problems: Systematic review of imaging studies. Alcohol and Alcoholism. 2013;48:433-444.
62. Brumback T, Squeglia LM, Jacobus J, Pulido C, Tapert SF, Brown SA. Adolescent heavy drinkers' amplified brain responses to alcohol cues decrease over one month of abstinence. Addictive Behaviors. 2015;46:45-52.
63. Lisdahl KM, Thayer R, Squeglia LM, McQueeny T, Tapert SF. Recent binge drinking predicts smaller cerebellar volumes in adolescents. Psychiatry Research: Neuroimaging. 2013;211:17-23.
64. McHugh ML. Interrater reliability: The kappa statistic. Biochemia Medica. 2012;22:276-282.
65. Veritas Health Innovation. Covidence systematic review software. Mebourne, Australia.
66. Clarivate Analytics. EndNote. Philadelphia, United States.
67. Brozek JL, Akl EA, Alonso-Coello P, Lang D, Jaeschke R, Williams JW, et al. Grading quality of evidence and strength of recommendations in clinical practice guidelines. Part 1 of 3. An overview of the GRADE approach and grading quality of evidence about interventions. Allergy. 2009;64:669-77.
68. Shamseer L, Moher D, Clarke M, Ghersi D, Liberati A, Petticrew M, et al. Preferred reporting items for systematic review and meta-analysis protocols (PRISMA-P) 2015: Elaboration and explanation. BMJ. 2015;349:g7647.

For peer review only

Figures and Tables

Figure 1: Logic model: Antecedents and consequences of binge drinking in adolescents and young adulthood.

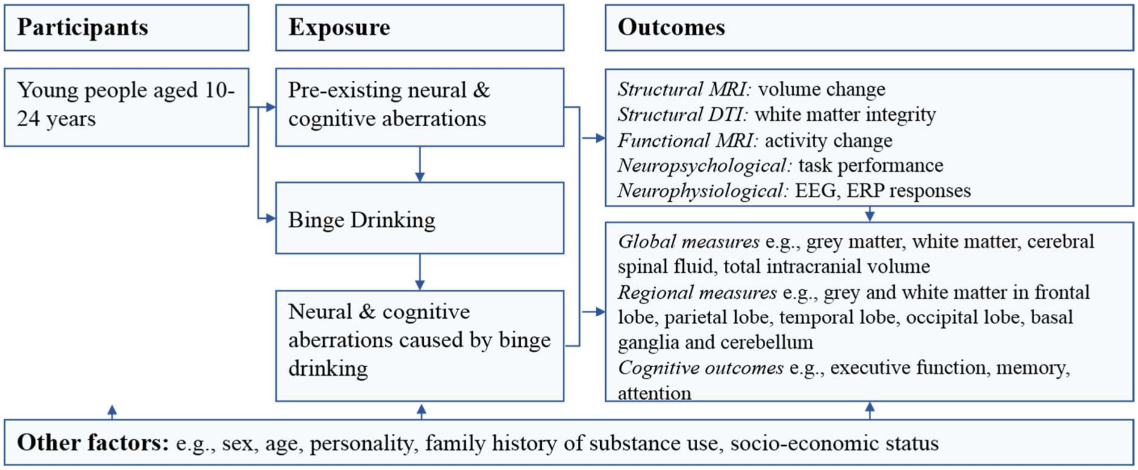

For peer review only

Table 1: Selection criteria

**Inclusion Criteria**	
Population	
1	Participants aged 10-24 years at first assessment
2	Study is available in the English language
3	N > 12 participants per group
4	Human participants (no animal studies)
Exposure	
5	Inclusion of binge drinking sample
Comparator	
6	Inclusion of a control group who do not meet criteria for binge drinking
Outcomes	
7	Use of neuropsychological, neurophysiological, structural or functional imaging techniques
8	Presentation of main effects
Study characteristics	
9	Peer-reviewed study
10	Cross-sectional or longitudinal data
11	Empirical data
12	Published before 1 April 2018
**Exclusion Criteria**	
Exposure	
13	Studies that involved participants who met criteria for alcohol use disorder
14	Studies that involved participants who were significantly involved with substances other than alcohol
15	Studies that involved participants who had other clinically diagnosed psychiatric, neurological or pharmacological conditions
Study characteristics	
16	Reviews, information in books, letters

Authors' Contributions

BL conceptualized the study and is the guarantor of the review. BL, LM, LS developed the study design and protocol. BL wrote the first draft of the manuscript. All authors read, revised and approved the final manuscript.

Funding statement

The first author is funded by the University of New South Wales and received a University of New South Wales Scientia PhD Scholarship. This research received no specific grant from any funding agency in the public, commercial or not-for-profit sectors.

Competing interests

None declared.

Supplement Files

1. PRISMA-P (Preferred Reporting Items for Systematic review and Meta-Analysis Protocols) 2015 checklist: recommended items to address in a systematic review protocol.
2. Search strategy for Medline.

For peer review only

Supplement File 2: Search strategy for Medline

Search	Terms
1.	(alcohol OR binge OR binge drink* OR ethanol).mp
2.	(neuroimage* OR brain OR brain imag* OR magnetic resonance OR functional magnetic resonance OR diffusion tensor imag* OR MRI OR fMRI OR DTI OR neuropsycholog* OR neurophysiolog* OR electrophysiolog* OR EEG OR ERP OR cogniti* OR verbal working memory OR episodic memory OR visuospatial working memory OR verbal fluency OR executive function OR digit symbol substitution OR reaction time OR attention).mp.
3.	(adol* OR youth OR emerging adult OR young adult OR teen*).mp.
5.	Limit 1-3 to human
6.	Limit 1-4 to English language
7.	Limit 1-5 to “Article” [Publication Type]

[mp=title, abstract, original title, name of substance word, subject heading word, keyword heading word, protocol supplementary concept word, rare disease supplementary concept word, unique identifier, synonyms].

For peer review only

Supplement File 1: PRISMA-P (Preferred Reporting Items for Systematic review and Meta-Analysis Protocols) 2015 checklist: recommended items to address in a systematic review protocol* [(68)]

Section and topic	Item No	Checklist item	Information reported
**ADMINISTRATIVE INFORMATION**			
Title:			
Identification	1a	Identify the report as a protocol of a systematic review	☒
Update	1b	If the protocol is for an update of a previous systematic review, identify as such	☐
Registration	2	If registered, provide the name of the registry (such as PROSPERO) and registration number	☒
Authors:			
Contact	3a	Provide name, institutional affiliation, e-mail address of all protocol authors; provide physical mailing address of corresponding author	☒
Contributions	3b	Describe contributions of protocol authors and identify the guarantor of the review	☒
Amendments	4	If the protocol represents an amendment of a previously completed or published protocol, identify as such and list changes; otherwise, state plan for documenting important protocol amendments	☐
Support:			
Sources	5a	Indicate sources of financial or other support for the review	☒
Sponsor	5b	Provide name for the review funder and/or sponsor	☐
Role of sponsor or funder	5c	Describe roles of funder(s), sponsor(s), and/or institution(s), if any, in developing the protocol	☐
**INTRODUCTION**			
Rationale	6	Describe the rationale for the review in the context of what is already known	☒
Objectives	7	Provide an explicit statement of the question(s) the review will address with reference to participants, interventions, comparators, outcomes and study characteristics (PICOS)	☒
**METHODS**			
Eligibility criteria	8	Specify the study characteristics (such as PICOS, study design, setting, time frame) and report characteristics (such as years considered, language, publication status) to be used as criteria for eligibility for the review	☒
Information sources	9	Describe all intended information sources (such as electronic databases, contact with study authors, trial registers or other grey literature sources) with planned dates of coverage	☒
Search strategy	10	Present draft of search strategy to be used for at least one electronic database, including planned limits, such that it could be repeated	☒
Study records:			
Data management	11a	Describe the mechanism(s) that will be used to manage records and data throughout the review	☒
Selection process	11b	State the process that will be used for selecting studies (such as two independent reviewers) through each phase of the review (that is, screening, eligibility and inclusion in meta-analysis)	☒
Data collection process	11c	Describe planned method of extracting data from reports (such as piloting forms, done independently, in duplicate), any processes for obtaining and confirming data from investigators	☒
Data items	12	List and define all variables for which data will be sought (such as PICOS items, funding sources), any pre-planned data assumptions and simplifications	☒
Outcomes and	13	List and define all outcomes for which data will be sought, including	☒

For peer review only

prioritization		prioritization of main and additional outcomes, with rationale	
Risk of bias in individual studies	14	Describe anticipated methods for assessing risk of bias of individual studies, including whether this will be done at the outcome or study level, or both; state how this information will be used in data synthesis	☒
Data synthesis	15a	Describe criteria under which study data will be quantitatively synthesised	☒
	15b	If data are appropriate for quantitative synthesis, describe planned summary measures, methods of handling data and methods of combining data from studies, including any planned exploration of consistency (such as I^2, Kendall's τ)	☒
	15c	Describe any proposed additional analyses (such as sensitivity or subgroup analyses, meta-regression)	☐
	15d	If quantitative synthesis is not appropriate, describe the type of summary planned	☒
Meta-bias(es)	16	Specify any planned assessment of meta-bias(es) (such as publication bias across studies, selective reporting within studies)	☐
Confidence in cumulative evidence	17	Describe how the strength of the body of evidence will be assessed (such as GRADE)	☒

*** It is strongly recommended that this checklist be read in conjunction with the PRISMA-P Explanation and Elaboration (cite when available) for important clarification on the items. Amendments to a review protocol should be tracked and dated. The copyright for PRISMA-P (including checklist) is held by the PRISMA-P Group and is distributed under a Creative Commons Attribution Licence 4.0.**

For peer review only

BMJ Open

Binge drinking in young people: Protocol for a systematic review of neuropsychological, neurophysiological and neuroimaging studies

Journal:	*BMJ Open*
Manuscript ID	bmjopen-2018-023629.R1
Article Type:	Protocol
Date Submitted by the Author:	17-Jun-2018
Complete List of Authors:	Lees, Briana; University of New South Wales, NHMRC Centre of Research Excellence in Mental Health and Substance Use, National Drug and Alcohol Research Centre<br>Mewton, Louise; University of New South Wales, NHMRC Centre of Research Excellence in Mental Health and Substance Use, National Drug and Alcohol Research Centre<br>Stapinski, Lexine; University of New South Wales, NHMRC Centre of Research Excellence in Mental Health and Substance Use, National Drug and Alcohol Research Centre<br>Squeglia, Lindsay; Medical University of South Carolina, Department of Psychiatry and Behavioral Sciences<br>Rae, Caroline; University of New South Wales, Neuroscience Research Australia<br>Teesson, Maree; University of New South Wales, NHMRC Centre for Research Excellence in Mental Health and Substance Use, National Drug and Alcohol Research Centre
**Primary Subject Heading**:	Research methods
Secondary Subject Heading:	Neurology
Keywords:	binge drinking, alcohol use, cognitive development, neurodevelopment, Child & adolescent psychiatry < PSYCHIATRY, young adult

SCHOLARONE™
Manuscripts

Title: Binge drinking in young people: Protocol for a systematic review of neuropsychological, neurophysiological and neuroimaging studies

Authors: Lees, B.[1 2], Mewton, L.[1 2], Stapinski, L. [1 2], Squeglia, L. M. [3], Rae, C. [4] & Teesson, M. [1 2]

[1] *NHMRC Centre of Research Excellence in Mental Health and Substance Use*
[2] *National Drug and Alcohol Research Centre, University of New South Wales, Sydney, NSW, Australia*
[3] *Department of Psychiatry and Behavioral Sciences, Medical University of South Carolina, Charleston, SC, United States*
[4] *Neuroscience Research Australia, University of New South Wales, Sydney, NSW, Australia*

Keywords: binge drinking, alcohol use, cognitive development, neurodevelopment, adolescence, young adult

Corresponding Author:
Briana Lees, BPsych (Hons)
National Drug and Alcohol Research Centre
University of New South Wales
National Drug and Alcohol Research Centre
22-32 King St
Randwick, 2031
Australia
Phone 61 (2) 8936 1012
Email: b.lees@unsw.edu.au

Word count: 3,583

For peer review only

Abstract

Introduction: Binge drinking is the most common pattern of alcohol use among young people in Western countries. Adolescence and young adulthood is a vulnerable developmental period and binge drinking during this time has a higher potential for neurotoxicity and interference with ongoing neural and cognitive development. The purpose of this systematic review will be to assess and integrate evidence of the impact of binge drinking on cognition, brain structure, and function in youth aged 10 to 24 years. Cross-sectional studies will synthesise the aberrations associated with binge drinking, while longitudinal studies will distinguish the cognitive and neural antecedents from the cognitive and neural effects that are a consequence of binge drinking.

Methods and analysis: A total of five peer-reviewed databases (PubMed, EMBASE, Medline, PsychINFO, ProQuest) will be systematically searched and the search period will include all studies published prior to 1 April 2018. The search terms will be a combination of MeSH keywords that are based on previous relevant reviews. Study selection will follow the Preferred Reporting Items for Systematic Reviews and Meta-Analyses (PRISMA) guidelines and study quality will be assessed using The Grades of Recommendation, Assessment, Development and Evaluation (GRADE) approach. All studies will be screened against eligibility criteria designed to synthesise studies that examined a young binge drinking sample and used neuropsychological, neurophysiological, or neuroimaging assessment techniques. Studies will be excluded if participants were significantly involved in other substances or if they had been clinically diagnosed with an alcohol use disorder, or any psychiatric, neurological, or pharmacological condition. If available data permits, a meta-analysis will be conducted.

Ethics and dissemination: Formal ethics approval is not required as primary data will not be collected. The results will be disseminated through a peer-reviewed publication, conference presentations and social media.

Trial registration number International Prospective Register for Systematic Reviews (PROSPERO) number: CRD42018086856.

Strengths and limitations of this study

- This systematic review and meta-analysis will be the first to synthesise neuropsychological, neurophysiological, and neuroimaging studies examining the developmental impact of binge drinking on cognition, brain structure, and function in youth.
- This review will report on cross-sectional and longitudinal data to firstly identify the cognitive and neural aberrations associated with binge drinking, and secondly to distinguish the antecedents of binge drinking from the effects that may be caused by binge drinking.

For peer review only - http://bmjopen.bmj.com/site/about/guidelines.xhtml

- Identified cognitive and neural precursors and consequences of binge drinking will be informative for prevention, early intervention, and treatment efforts.
- While studies will be excluded if participants had been clinically diagnosed with an alcohol use disorder, mild alcohol dependence that has not been formally diagnosed may be more prevalent in adolescent binge drinkers, and this may increase the risk of bias in the review towards a binge population that was seeking help or treatment.

For peer review only

For peer review only - http://bmjopen.bmj.com/site/about/guidelines.xhtml

Introduction

Alcohol misuse amongst young people is widely recognised as a global health priority [1] and has raised concern about the neurotoxic effects of alcohol use on a large scale [2]. Binge drinking is a pattern of alcohol use that brings blood alcohol concentration levels to 0.08 g/dL which typically occurs after the consumption of four or more alcoholic drinks per drinking occasion (i.e., at the same time or within a couple of hours of each other) for females and five or more drinks per occasion for males [3, 4]. This episodic pattern of drinking, where an individual drinks less frequently but in larger amounts, is most common among adolescents in Western countries [5-7]. For instance, approximately 10-16% (United States; US), 23% (United Kingdom) and 15% (Australia) of 15-17 year olds report binge drinking in the previous month [8-10]. The prevalence of binge drinking sharply increases from adolescence to young adulthood, with 40-50% of young adults reporting binge drinking at least monthly [6, 9, 11-13] . Extreme binge drinking (defined as 10 or more drinks per occasion) is also common, with 29% of young adults in Australia and 16% of US adolescents engaging in this behaviour [9, 14, 15]. This is concerning because early alcohol use and binge drinking is associated with a myriad of short- and long-term negative consequences including blackouts, hangovers and alcohol poisoning [16, 17], alcohol and drug use disorders [18-20], other mental health problems [21], risky sexual behaviours [22, 23], injuries [24, 25] and increased risk of being a victim of assault or accidental death [26, 27].

Studies consistently indicate that alcohol use and misuse during adolescence (10-19 years) and young adulthood (20-24 years) has a higher potential for neurotoxicity and interference with ongoing neural and cognitive development than during later adulthood [16, 25, 28-36]. This is because adolescence and young adulthood is a vulnerable developmental period characterised by significant neural changes. Although brain size is thought to stabilise around the age of five years [37], important morphometric restructuring and functional neuromaturation continues to occur during adolescence and young adulthood with substantial myelinisation, synaptic refinement, and changes in grey and white matter volume until the age of 25 years [38]. The reward and mesolimbic systems mature during mid-adolescence, prior to the development of prefrontal and cognitive control regions which continue to develop into late adolescence [39-42]. This has a two-fold effect, firstly this hypersensitivity to reward during adolescence results in an increased propensity to engage in risky and sensation-seeking activities, including drug and alcohol use [43]. Secondly, risky drinking during prefrontal brain development may interfere with neuromaturation and translate to ongoing neural aberrations and top-down cognitive processing deficits, reducing youth's ability to enable self-control and resist temptations (inhibition); to see reason, problem solve, and consider alternatives (working memory);

For peer review only - http://bmjopen.bmj.com/site/about/guidelines.xhtml

and to plan and change perspective (cognitive flexibility) [44]. Collectively, these changes in cognitive processing may lead to increased motivation to consume alcohol and a decreased ability to regulate this motivation and drinking behaviour. As many of these developmental changes occur in brain regions that appear to be particularly sensitive to alcohol [45, 46], it is critical that research examines the associated negative consequences of risky episodic drinking during a vulnerable developmental period as the neural insults may have ongoing cognitive and behavioural impacts.

The growing concern about alcohol use amongst young people has led to a significant increase in the number of studies using neuropsychological, neurophysiological, and neuroimaging techniques to determine the effects on brain and cognitive development. Over the past decade there has also been a rise in the number of longitudinal designs that assess young people before they initiate alcohol use and continue to assess them over time as a portion begin to initiate use. These prospective longitudinal studies have made it possible to disentangle the antecedents and consequences of alcohol use in young people. A recent review of longitudinal studies that concentrated on alcohol initiation in adolescence found that reduced grey matter volume (frontal), less white matter volume (cerebellar, nucleus accumbens, anterior cingulate), poor white matter integrity (fronto-limbic), decreased activation during inhibition and working memory tasks, and increased reward response (frontal) were antecedents of alcohol use initiation in adolescence [47]. Accelerated decreases in grey matter (frontal, temporal), attenuated white matter development (pons, corpus callosum), poor white mater integrity, and increased brain activation during inhibition and working memory tasks were reported consequences following alcohol use initiation. In terms of cognitive domains, poorer inhibitory functioning and working memory were antecedents of alcohol initiation in adolescence while poorer verbal learning and memory, visuospatial functioning, psychomotor speed and working memory were reported effects following alcohol use initiation in adolescence. By distinguishing the antecedents from the consequences of alcohol use initiation, this review provides researchers with specific neural and cognitive domains to target in prevention and treatment efforts.

Considering binge drinking is the dominant pattern of use among young people, it is important to understand the neural and cognitive impact this pattern of drinking has on the developing brain. Several narrative reviews (summarising neuropsychological, neurophysiological, and neuroimaging studies; see [7, 17, 28, 45, 48, 49]) and one systematic review from 2014 (including only neuroimaging studies [50]) have summarised the recent binge drinking literature. The systematic review concluded

For peer review only - http://bmjopen.bmj.com/site/about/guidelines.xhtml

that there were a number of structural changes associated with binge drinking, including smaller grey and white matter volume compared to non-binge drinkers [29, 51] and lower white matter integrity across more than 18 white matter regions [52, 53]. Functional differences reported in binge drinkers included less activation during a spatial working memory task [54] and abnormal activation during verbal encoding [55] and decision-making tasks [56]. In terms of neuropsychological studies, narrative reviews have concluded that binge drinking is associated with several cognitive deficits, including impairments in verbal, non-verbal and spatial working memory, as well as attention and executive function [33-35, 57]. A review of neurophysiological studies found that young binge drinkers displayed latency differences in several ERP components, including P1, N1, P3, P3b and P450, in response to a number of cognitive functioning tasks [28]. The early positive and negative voltage deflections (P1, N1) reflect initial sensory differences between binge drinkers and non-binge drinkers, while the later components (P3, P3b, P450) reflect differences in the way participants processed the cognitive tasks. Overall, there is a growing evidence base that consistently demonstrates neural and cognitive aberrations associated with binge drinking. More recently, there has been an increase in the number of prospective longitudinal studies examining the effect of binge drinking among young people. Integrating these new findings is essential to understanding whether the neural and cognitive precursors and consequences of binge drinking are similar or divergent to the domains related to alcohol initiation in adolescence.

The aim of this systematic review is to therefore provide an update on the expanding literature and synthesise the neuropsychological, neurophysiological, and neuroimaging literature on binge drinking and neurodevelopment. This review will also address limitations identified in the previous systematic review. The authors of the 2014 systematic review limited their search to one peer-reviewed database, included adolescents aged 10-19 years and included concurrent substance use. Searching a broader range of peer-reviewed databases may identify studies which were potentially missed in the previous review. Expanding the age range to include young people aged 10-24 years aligns with evidence that neuromaturation continues into the mid-20s [38], as well as the World Health Organisation's definition of young people [58]. To examine the specific effect of binge drinking on brain development and functioning, studies should exclude individuals with concurrent regular use of drugs other than alcohol [45, 47] as well as exclude samples that characterised drinking based on non-binge, heavy-drinking or diagnostic criteria, including alcohol abuse, dependence and alcohol use disorder, due to the heterogeneity of drinking behaviours and related harms [49]. Alcohol use disorder is characterised by continued alcohol use despite clinically significant social and physiological consequences, including substance abuse, affective symptoms and other

For peer review only - http://bmjopen.bmj.com/site/about/guidelines.xhtml

psychopathology [59]. Therefore, the type, extent and magnitude of the neural and cognitive aberrations associated with alcohol use disorder are likely to differ from those associated with an adolescent, socially functioning binge drinking population. Additionally, no review has systematically integrated neuropsychological, neurophysiological, and neuroimaging data. Integrating neuropsychological and neurophysiological studies with neuroimaging research is crucial because cognitive processes make an important contribution to excessive alcohol consumption [57] and assessing this data conjointly will provide a broader understanding of the impact binge drinking has on brain development and behaviour. Finally, previous reviews have not critically appraised the within-study risk of bias or overall quality of the body of literature.

This review will involve conducting a systematic literature search of cross-sectional and longitudinal studies to assess and integrate the evidence regarding the impact of binge drinking on cognition, brain structure, and function, utilising neuropsychological, neurophysiological (electroencephalography; EEG, event-related potential; ERP) and neuroimaging (magnetic resonance imaging; MRI, functional MRI; fMRI, diffusion tensor images; DTI, magnetic resonance spectroscopy; MRS) studies. If available data permits, a meta-analysis will be conducted to determine the overall effects of binge drinking on the outcomes of interest. By including cross-sectional studies, we aim to synthesise the cognitive and neural aberrations associated with binge drinking in young people. On the other hand, longitudinal studies that track individuals over time will distinguish cognitive and neural antecedents that predict later binge drinking from the cognitive and neural effects that are a consequence of binge drinking during adolescence and young adulthood (see Figure 1 for logic model). Due to the limited number of published longitudinal studies at the time of the previous systematic review, this systematic review will be the first to infer causality. The predisposing and consequential factors may not be mutually exclusive and some of the vulnerability factors that predict binge drinking behaviour may also be further impacted by the initiation and continuation of binge drinking. Importantly, identified precursors of binge drinking will be informative for prevention and early intervention efforts. Meanwhile, by identifying consequences of binge drinking, treatment efforts will be able to pursue targeted cognitive and physiological training to determine whether these neural insults have ongoing cognitive and behavioural impacts or whether they can recover following a decrease in alcohol use.

[Insert Figure 1 approximately here]

For peer review only - http://bmjopen.bmj.com/site/about/guidelines.xhtml

Objectives

1. To assess and integrate evidence of the impact of binge drinking on cognitive, structural, and functional development in people aged 10-24 years, compared to healthy controls who do not meet the criteria for binge drinking.
2. To synthesise the cognitive and neural aberrations associated with binge drinking by utilising cross-sectional data.
3. To identify the cognitive, structural, and functional features that predispose youth to binge drinking, and separate this from the cognitive, structural and functional features that may be a consequence of binge drinking.
4. To examine the within-study risk of bias and assess the quality of the body of evidence examining the relationship between binge drinking and cognitive, structural, and functional deficits in adolescents and young adults.

Methods

This protocol follows the Preferred Reporting Items for Systematic Reviews and Meta-Analysis Protocols (PRISMA-P) statement [(60)], found in the Supplement File. This protocol has been registered with the PROSPERO International Prospective Register of Systematic Reviews of the University of York (registration number: CRD42018086856).

Search Strategy

Relevant literature from PubMed, EMBASE, Medline, PsycINFO and ProQuest will be systematically searched using a comprehensive search strategy which was developed using medical subject headings (MeSH). The Ovid Medline search strategy is provided in the Supplement File, which will be replicated for the other electronic databases. This strategy will search through all relevant literature published from database inception to 1 April 2018. A snowballing technique will be employed where the reference list of identified articles will also be screened for suitable studies.

Search terms will be used to identify neuropsychological, neurophysiological, and neuroimaging studies assessing the impact of binge drinking on neurodevelopment and neuropsychological task performance in adolescents and young adults. The search terms will be based on previous reviews examining the association between substance use, cognition, brain structure, and function [(16, 50, 61, 62)]. Search terms will be combinations of MeSH keywords describing the participants (adolescent,

For peer review only - http://bmjopen.bmj.com/site/about/guidelines.xhtml

teenager, youth, emerging adult, young adult), the exposure variable (alcohol, binge drinking, ethanol), and the assessment methods measuring the outcomes of interest (neuroimaging, brain imaging, MRI, fMRI, DTI, MRS, neurophysiological, EEG, ERP, neuropsychological, cognitive, verbal working memory tests, episodic memory tests, visuospatial working memory tests, verbal fluency tests, executive function tests, digit symbol substitution tests, reaction time, attention). Two reviewers will be involved in independently screening articles, extracting data and assessing the methodological quality.

Eligibility Criteria

Eligibility criteria for this review are defined using PICOS (population, intervention/exposure, comparator, outcome, study characteristics). Table 1 provides an overview of the selection criteria.

Table 1: Selection criteria

**Inclusion Criteria**	
Population	
1	Participants aged 10-24 years at first assessment
2	Study is available in the English language
3	N > 12 participants per group
4	Human participants (no animal studies)
Exposure	
5	Inclusion of binge drinking sample
Comparator	
6	Inclusion of a control group who do not meet criteria for binge drinking
Outcomes	
7	Use of neuropsychological, neurophysiological, structural or functional imaging techniques
8	Presentation of main effects
Study characteristics	
9	Peer-reviewed study
10	Cross-sectional or longitudinal data
11	Empirical data
12	Published before 1 April 2018
**Exclusion Criteria**	
Exposure	
13	Studies that involved participants who met criteria for alcohol use disorder
14	Studies that involved participants who were significantly involved with substances other than alcohol
15	Studies that involved participants who had other clinically diagnosed psychiatric, neurological or pharmacological conditions
Study characteristics	
16	Reviews, information in books, letters

Population

Study samples will be limited by age to human adolescents and young adults ranging from 10 to 24 years at first assessment, which is consistent with the World Health Organisation's definition of young people [(58)]. Studies that include both a sample of young people and an adult sample (>24

For peer review only - http://bmjopen.bmj.com/site/about/guidelines.xhtml

years) will be included if the majority of participants are aged 10 to 24 years or if a separate analysis for participants within the age range of this review was provided. A minimum of 12 participants per group (binge, comparator) must be included within the study analysis, consistent with previous reviews in this area [50]. Finally, studies must be available in the English language to be included in this review.

Exposure

Studies must include a binge drinking sample, where binge drinking is defined as four or more drinks per occasion for females or five or more drinks per occasion for males [3, 4]. Consistent with previous reviews in this area, studies will not be included in this review if samples have ever met diagnostic criteria (e.g., alcohol abuse or alcohol use disorder) [49], or if majority of the participants were significantly involved with substances other than alcohol (i.e., >5 cannabis use per month, >25 lifetime other drug use occasions [54, 63, 64]). It is noted that mild alcohol dependence that has not been clinically diagnosed may be more prevalent in adolescent or young adult binge drinkers, and this may result in an increased risk of bias in the review towards a binge drinking population that was seeking help or treatment compared to binge drinkers who were not. Studies that include participants who smoke tobacco will be included. Participant disclosure of other substance use or a urine sample identifying other substance use will be sufficient to exclude these studies. Studies that included participants who had other clinically diagnosed psychiatric, neurological, or pharmacological conditions will also be excluded from this review to ensure that outcomes are specific to binge drinking.

Comparator

Inclusion of a control group is required for studies to be included in this review. Studies must compare participants who meet the criteria for binge drinking with healthy controls who have never consumed alcohol or who have consumed low levels of alcohol but have never met the criteria for binge drinking.

Outcomes

Studies must report empirical data where the primary outcomes of interest are global and regional volume (structural images), global and regional activity (functional images; cerebral blood flow, regional cerebral blood flow, or blood oxygen level dependent signal), white matter integrity (DTI), neurochemical activity (MRS; glutamate, gamma-aminobutyric acid, *N*-acetylaspartate), brain

For peer review only - http://bmjopen.bmj.com/site/about/guidelines.xhtml

electrical activity (EEG, ERP responses), and cognitive task performance in neuropsychological and fMRI studies. Global measures include grey matter, white matter, cerebral spinal fluid, and total intracranial volume differences between the active and control group. Regional measures include white matter and grey matter (frontal lobe, parietal lobe, temporal lobe, occipital lobe, basal ganglia and cerebellum) differences between the active and control group. For neuroimaging and neurophysiological studies, detailed results of significant findings will be reported. For neuropsychological studies, significant differences in cognitive task performance between the active and control group will be reported.

Study characteristics

Peer-reviewed cross-sectional and longitudinal neuropsychological, neurophysiological, and neuroimaging studies that provide original data and were published before 1 April 2018 will be included. Reviews and information in books or letters will not be included. Any publication that reported data using two or more techniques from the same subject (e.g. structural MRI and functional MRI) will be considered separately in the review.

Selection procedure

Two researchers will be involved in the review and selection procedure. Reviewer one (BL) will screen all titles and abstracts from the peer-reviewed databases to determine eligibility for inclusion in the review. Reviewer two (LM) will independently screen a random selection of 25% of abstracts to ensure accuracy in the study selection. Cohen's kappa will be calculated to assess the inter-rater agreement between the two reviewers. To ensure a strong level of agreement, a Cohen's kappa of at least 0.8 is required [65]. Full-text versions of the potentially eligible studies will be assessed by both reviewers to further determine eligibility for inclusion. Again, Cohen's kappa will be calculated at the full-text screening stage. Consultation between reviewers will be held at the time of abstract screening and full-text assessment to reconcile any differences of opinion. If consensus cannot be reached, a third member of the research team (LS) will review the eligibility of the study.

Data extraction

All citations will be imported into Covidence [66] and Endnote [67]. Endnote will be used to store and manage all review data. Covidence will be used to screen titles, abstracts and full texts. Reviewer one will extract data using a data extraction spreadsheet in Excel. Study characteristics will be

For peer review only - http://bmjopen.bmj.com/site/about/guidelines.xhtml

extracted from published papers, with study authors contacted in the event of missing data. The following information will be extracted from the included studies:

1. *Study information:* names of authors, year of publication, primary outcome measurements, statistical approaches;
2. *Participant characteristics:* sample size, sex, age, handedness, other substance use (i.e., tobacco use, cannabis use that is <5 occasions/month);
3. *Alcohol characteristics:* age of onset, frequency of binge drinking, mean quantity of alcohol consumed;
4. *Study characteristics:* imaging modality and analysis, binge drinking sample and control group criteria, cognitive task performed, cognitive and neural domain measured, neurophysiological activity measured, rest/active condition (for functional imaging studies), and exclusion criteria, including absence of neurological, psychiatric, or pharmacological conditions, alcohol use disorder or significant involvement with substances other than alcohol; and
5. *Results:* results of outcomes of interest for this review.

Data analysis and quality assessment

A table summarising the results will be produced, including information about imaging modality and analysis or neuropsychological tests, sample information, alcohol characteristics, and the study findings. For longitudinal studies, pre-existing cognitive, structural, and functional features will be separated from cognitive, structural, and functional features that are evident as a consequence of binge drinking. If available data permits, a meta-analysis will be conducted using Comprehensive Meta-Analysis. Hedges' g will be calculated to determine the binge drinking between-group standardised mean effect size from outcomes of interest (global and regional volume, white matter integrity, neural activity, and cognitive performance). A random effects model will be adopted as wide variations in participant characteristics and methodological factors are expected between the studies.

In the case of insufficient homogenous data, a narrative synthesis of the main results extracted from the studies will be completed. The studies will be classified according to the study type (i.e., neuropsychological task, neurophysiological measurement, structural imaging, functional imaging) and a summary of differences identified in the binge drinking sample compared to the control group will be reported in text.

For peer review only - http://bmjopen.bmj.com/site/about/guidelines.xhtml

Following data extraction, the quality of each study will be critically appraised using The Grades of Recommendation, Assessment, Development, and Evaluation (GRADE) approach [68]. The GRADE system entails an assessment of the quality of a body of evidence for each individual outcome. The GRADE approach defines the quality of the body evidence as the extent to which one can be confident that an estimate of effect or association is close to the quantity of specific interest. This involves considerations of within-study risk of bias (methodological quality of design), directness of evidence, heterogeneity of results, precision of results, and the probability of publication bias. Reviewer one will critically appraise all included studies using the GRADE system. Reviewer two will assess the quality of a random selection of 25% of studies to ensure scoring accuracy. Consultation between reviewers will be held to reconcile any differences of opinion.

Patient and Public Involvement

Patients and the public were not involved in this systematic review protocol.

Ethics and Dissemination

Ethical approval is not required for this study. The systematic review will be published in a peer-reviewed journal, presented at conferences and will be shared on social media platforms.

Conclusion

This paper summarises the protocol for a systematic review of neuropsychological, neurophysiological, and neuroimaging studies conducted in youth who binge drink. The purpose of this review is to assess and integrate the evidence of the developmental impact of binge drinking on cognition, brain structure, and function. Cross-sectional studies will be included in order to synthesise the cognitive and neural aberrations associated with binge drinking in young people. Longitudinal data will be sought to distinguish cognitive and neural antecedents of binge drinking from the cognitive and neural effects that are a consequence of binge drinking during adolescence and young adulthood. This review will be the first to synthesise neuropsychological, neurophysiological, and neuroimaging evidence in a systematic way, to include a meta-analysis of the findings, and the first to assess the quality of the body of neuropsychological, neurophysiological and neuroimaging studies. This review aims to provide researchers, policy makers and program developers with identified antecedents and consequences of binge drinking that will be informative for prevention, early intervention and treatment efforts.

For peer review only - http://bmjopen.bmj.com/site/about/guidelines.xhtml

References

1. World Health Organization. Global status report on alcohol and health. Geneva; 2014.
2. Patrick ME, Terry-McElrath YM, Kloska DD, Schulenberg JE. High-intensity drinking among young adults in the United States: Prevalence, frequency, and developmental change. Alcoholism, clinical and experimental research. 2016;40:1905-1912.
3. National Institute on Alcohol Abuse and Alcoholism (NIAAA). Drinking levels defined. 2018; https://www.niaaa.nih.gov/alcohol-health/overview-alcohol-consumption/moderate-binge-drinking (Accessed 12 April 2018).
4. Substance Abuse and Mental Health Services Administration (SAMHSA). Binge drinking: Terminology and patterns of use. 2016; https://www.samhsa.gov/capt/tools-learning-resources/binge-drinking-terminology-patterns (Accessed 12 April 2018).
5. Substance Abuse and Mental Health Services Administration (SAMHSA). Results from the 2008 national survey on drug use and health: Summary of national findings. Rockville, MD: Office of Applied Studies; 2009.
6. Archie S, Zangeneh Kazemi A, Akhtar-Danesh N. Concurrent binge drinking and depression among Canadian youth: Prevalence, patterns, and suicidality. Alcohol. 2012;46:165-72.
7. Hermens DF, Lagopoulos J, Tobias-Webb J, De Regt T, Dore G, Juckes L, et al. Pathways to alcohol-induced brain impairment in young people: A review. Cortex. 2013;49:3-17.
8. Windle M. Drinking over the lifespan: Focus on early adolescents and youth. Alcohol Research: Current Reviews. 2016;38:95-101.
9. Australian Institute of Health and Welfare (AIHW). National drug strategy household survey 2016: Detailed findings. Canberra, ACT: AIHW; 2017.
10. Johnston LD, O'Malley PM, Miech RA, Bachman JG, Schulenberg JE. Monitoring the Future national survey results on drug use, 1975-2017: Overview, key findings on adolescent drug use. Ann Arbor: Institute for Social Research, The University of Michigan; 2017.
11. Substance Abuse and Mental Health Services Adminstration (SAMHSA). Results from the 2010 national survey on drug use and health: Summary of national findings. Rockville, MD: Office of Applied Studies; 2011.
12. Australian Institute of Health and Welfare (AIHW). National drug strategy household survey detailed report 2013. Canberra, ACT: AIHW; 2014.
13. Schuckit MA, Smith TL, Heron J, Hickman M, Macleod J, Munafo MR, et al. Latent trajectory classes for alcohol-related blackouts from age 15 to 19 in ALSPAC. Alcoholism Clinical & Experimental Research. 2015;39:108-116.
14. Eaton DK, Kann L, Kinchen S, Shanklin S, Flint KH, Hawkins J, et al. Youth risk behaviour surveillance - United States. Morbidity and Mortality Weekly Report Surveillance Summaries (Washington, DC: 2002). 2012;61:1-162.
15. Patrick ME, Schulenberg JE, Martz ME, Maggs JL, O'Malley PM, Johnston LD. Extreme binge drinking among 12th-grade students in the United States: Prevalence and predictors. JAMA Pediatrics. 2013;167:1019-1025.
16. Zeigler DW, Wang CC, Yoast RA, Dickinson BD, McCaffree MA, Robinowitz CB, et al. The neurocognitive effects of alcohol on adolescents and college students. Preventive Medicine. 2005;40:23-32.
17. Hermens DF, Lagopoulos J. Binge drinking and the young brain: A mini review of the neurobiological underpinnings of alcohol-induced blackout. Frontiers in Psychology. 2018;9:12.
18. Chassin L, Hussong A, Barrera MJ, Molina BSG, Trim R, Ritter J. Adolescent substance use. In: Lerner RM, Steinberg L, editors. Handbook of Adolescent Psychology (2nd ed). Hoboken, NJ, USA: John Wiley & Sons Inc.; 2004. p. 665-96.

For peer review only - http://bmjopen.bmj.com/site/about/guidelines.xhtml

19. Grant BF, Dawson DA. Age at onset of alcohol use and its association with DSM-IV alcohol abuse and dependence: Results from the National Longitudinal Alcohol Epidemiologic Survey. Journal of Substance Abuse. 1997;9:103-110.
20. Newton-Howes G, Boden JM. Relation between age of first drinking and mental health and alcohol and drug disorders in adulthood: Evidence from a 35-year cohort study. Addiction. 2016;111:637-644.
21. Welsh JW, Knight JR, Hou SS, Malowney M, Schram P, Sherritt L, et al. Association between substance use diagnoses and psychiatric disorders in an adolescent and young adult clinic-based population. Journal of Adolescent Health. 2017;60:648-52.
22. Halpern-Felsher BL, Millstein SG, Ellen JM. Relationship of alcohol use and risky sexual behaviour: A review and analysis of findings. Journal of Adolescent Health. 1996;19:331-336.
23. Tapert SF, Aarons GA, Sedlar GR, Brown SA. Adolescent substance use and sexual risk-taking behaviour. Journal of Adolescent Health. 2001;28:181-189.
24. Porter RS. Alcohol and injury in adolescents. Pediatric Emergency Care. 2000;16:316-320.
25. Spear LP. Adolescents and alcohol: Acute sensitivities, enhanced intake, and later consequences. Neurotoxicology and Teratology. 2014;41:51-59.
26. Bonomo YA, Bowes G, Coffey C, Carlin JB, Patton GC. Teenage drinking and the onset of alcohol dependence: A cohort study over seven years. Addiction. 2004;99:1520-8.
27. Mundt MP, Zakletskaia LI, Brown DD, Fleming MF. Alcohol-induced memory blackouts as an indicator of injury risk among college drinkers. Injury Prevention. 2012;18:44-49.
28. Jacobus J, Tapert SF. Neurotoxic effects of alcohol in adolescence. Annual Review of Clinical Psychology. 2013;9:703-21.
29. Lisdahl KM, Gilbart ER, Wright NE, Shollenbarger S. Dare to delay? The impacts of adolescent alcohol and marijuana use onset on cognition, brain structure and function. Frontiers in Psychiatry. 2013;4:53.
30. Peeters M, Vollebergh WA, Wiers RW, Field M. Psychological changes and cognitive impairments in adolescent heavy drinkers. Alcohol and Alcoholism. 2014;49:182-186.
31. Squeglia LM, Jacobus J, Tapert SF. The influence of substance use on adolescent brain development. Clinical EEG and Neuroscience. 2009;40:31-38.
32. Squeglia LM, Jacobus J, Tapert SF. The effect of alcohol use on human adolescent brain structures and systems. Handbook of Clinical Neurology. 2014;125:501-510.
33. Brown SA, Tapert SF, Granholm E, Delis DC. Neurocognitive functioning of adolescents: Effects of protracted alcohol use. Alcoholism Clinical & Experimental Research. 2000;24:164-171.
34. Tapert SF, Brown SA. Neuropsychological correlates of adolescent substance abuse: Four-year outcomes. Journal of the International Neuropsychological Society. 1999;5:481-493.
35. Tapert SF, Granholm E, Leedy NG, Brown SA. Substance use and withdrawal: Neuropsychological functioning over 8 years in youth. Journal of the International Neuropsychological Society. 2002;8:873-883.
36. Nixon K, McClain JA. Adolescence as a critical window for developing an alcohol use disorder: Current findings in neuroscience. Current Opinion in Psychiatry. 2010;23:227-232.
37. Durston S, Hulshoff Pol HE, Casey BJ, Giedd JN, Buitelaar JK, van Engeland H. Anatomical MRI of the developing human brain: What have we learned? Journal of the American Academy of Child and Adolescent Psychiatry. 2001;40:1012-1020.
38. Giedd JN. Structural magnetic resonance imaging of the adolescent brain. Annals of the New York Academy of Sciences. 2004;1021:77-85.
39. Casey BJ, Jones RM, Hare TA. The adolescent brain. Annals of the New York Academy of the Sciences. 2008;1124:111-126.
40. Ernst M, Nelson EE, Jazbec S, McClure EB, Monk CS, Leibenluft E, et al. Amygdala and nucleus accumbens in responses to receipt and omission of gains in adults and adolescents. NeuroImage. 2005;25:1279-1291.

For peer review only - http://bmjopen.bmj.com/site/about/guidelines.xhtml

41. Koob GF, Volkow ND. Neurocircuitry of addiction. Neuropsychopharmacology. 2010;35:217-238.
42. Sturman DA, Moghaddam B. Reduced neuronal inhibition and coordination of adolescent prefrontal cortex during motivated behavior. The Journal of Neuroscience. 2011;31:1471-1478.
43. Somerville LH, Jones RM, Casey BJ. A time of change: Behavioral and neural correlates of adolescent sensitivity to appetitive and aversive environmental cues. Brain Cognition. 2010;72:124-133.
44. Diamond A. Executive functions. Annual Review of Psychology. 2013;64:135-168.
45. Spear LP. Effects of adolescent alcohol consumption on the brain and behaviour. Nature Reviews Neuroscience. 2018;19:197-214.
46. Crews FT, Vetreno RP, Broadwater MA, Robinson DL. Adolescent alcohol exposure persistently impacts adult neurobiology and behavior. Pharmacological Reviews. 2016;68:1074-1109.
47. Squeglia LM, Gray KM. Alcohol and drug use and the developing brain. Current Psychiatry Reports. 2016;18:46.
48. Petit G, Maurage P, Kornreich C, Verbanck P, Campanella S. Binge drinking in adolescents: A review of neurophysiological and neuroimaging research. Alcohol and Alcoholism. 2014;49:198-206.
49. Cservenka A, Brumback T. The Burden of Binge and Heavy Drinking on the Brain: Effects on Adolescent and Young Adult Neural Structure and Function. Frontiers in Psychology. 2017;8:1111.
50. Feldstein Ewing SW, Sakhardande A, Blakemore S-J. The effect of alcohol consumption on the adolescent brain: A systematic review of MRI and fMRI studies of alcohol-using youth. NeuroImage: Clinical. 2014;5:420-37.
51. Luciana M, Collins PF, Muetzel RL, Lim KO. Effects of alcohol use initiation on brain structure in typically developing adolescents. American Journal of Drug and Alcohol Abuse. 2013;39:345-355.
52. Jacobus J, McQueeny T, Bava S, Schweinsburg BC, Frank LR, Yang TT, et al. White matter integrity in adolescents with histories of marijuana use and binge drinking. Neurotoxicology Teratology. 2009;31:349-355.
53. McQueeny T, Schweinsburg BC, Schweinsburg AD, Jacobus J, Bava S, Frank LR, et al. Altered white matter integrity in adolescent binge drinkers. Alcoholism, Clinical and Experimental Research. 2009;33:1278-1285.
54. Squeglia LM, Schweinsburg AD, Pulido C, Tapert SF. Adolescent binge drinking linked to abnormal spatial working memory brain activation: Differential gender effects. Alcoholism Clinical & Experimental Research. 2011;35:1831-1841.
55. Schweinsburg AD, McQueeny T, Nagel BJ, Eyler LT, Tapert SF. A preliminary study of functional magnetic resonance imaging response during verbal encoding among adolescent binge drinkers. Alcohol. 2010;44:111-117.
56. Xiao L, Bechara A, Gong Q, Huang X, Li X, Xue G, et al. Abnormal affective decision making revealed in adolescent binge drinkers using a functional magnetic resonance imaging study. Psychology of Addictive Behaviors. 2013;27:443-454.
57. Field M, Schoenmakers T, Wiers RW. Cognitive processes in alcohol binges: A review and research agenda. Current Drug Abuse Reviews. 2008;1:263-279.
58. World Health Organization (WHO). Recognizing adolescence. 2014; http://apps.who.int/adolescent/second-decade/section2/page1/recognizing-adolescence.html (Accessed 13 April 2018).
59. Mewton L, Teesson M, Slade T, Grove R. The epidemiology of DSM-IV alcohol use disorders amongst young adults in the Australian population. Alcohol and Alcoholism. 2011;46:185-191.

For peer review only - http://bmjopen.bmj.com/site/about/guidelines.xhtml

60. Moher D, Shamseer L, Clarke M, Ghersi D, Liberati A, Petticrew M, et al. Preferred reporting items for systematic review and meta-analysis protocols (PRISMA-P) 2015 statement. Systematic Reviews. 2015;4:1.
61. Batalla A, Bhattacharyya S, Yucel M, Fusar-Poli P, Crippa JA, Nogue S, et al. Structural and functional imaging studies in chronic cannabis users: A systematic review of adolescent and adult findings. PLoS ONE. 2013;8:e55821.
62. Welch KA, Carson A, Lawrie SM. Brain structure in adolescents and young adults with alcohol problems: Systematic review of imaging studies. Alcohol and Alcoholism. 2013;48:433-444.
63. Brumback T, Squeglia LM, Jacobus J, Pulido C, Tapert SF, Brown SA. Adolescent heavy drinkers' amplified brain responses to alcohol cues decrease over one month of abstinence. Addictive Behaviors. 2015;46:45-52.
64. Lisdahl KM, Thayer R, Squeglia LM, McQueeny T, Tapert SF. Recent binge drinking predicts smaller cerebellar volumes in adolescents. Psychiatry Research: Neuroimaging. 2013;211:17-23.
65. McHugh ML. Interrater reliability: The kappa statistic. Biochemia Medica. 2012;22:276-282.
66. Veritas Health Innovation. Covidence systematic review software. Mebourne, Australia.
67. Clarivate Analytics. EndNote. Philadelphia, United States.
68. Brozek JL, Akl EA, Alonso-Coello P, Lang D, Jaeschke R, Williams JW, et al. Grading quality of evidence and strength of recommendations in clinical practice guidelines. Part 1 of 3. An overview of the GRADE approach and grading quality of evidence about interventions. Allergy. 2009;64:669-77.

For peer review only

For peer review only - http://bmjopen.bmj.com/site/about/guidelines.xhtml

Figure legends

Figure 1: Logic model: Antecedents and consequences of binge drinking in adolescents and young adulthood.

Authors' Contributions

BL conceptualized the study and is the guarantor of the review. BL, LM, LS developed the study design and protocol. LMS, CR and MT provided feedback on the study design and protocol. BL wrote the first draft of the manuscript. All authors read, revised and approved the final manuscript.

Funding statement

The first author is funded by the University of New South Wales and received a University of New South Wales Scientia PhD Scholarship. This research received no specific grant from any funding agency in the public, commercial or not-for-profit sectors.

Competing interests

None declared.

Supplement Files

1. Supplementary Methods: PRISMA-P (Preferred Reporting Items for Systematic review and Meta-Analysis Protocols) 2015 checklist: recommended items to address in a systematic review protocol, search strategy for Medline.

For peer review only

For peer review only - http://bmjopen.bmj.com/site/about/guidelines.xhtml

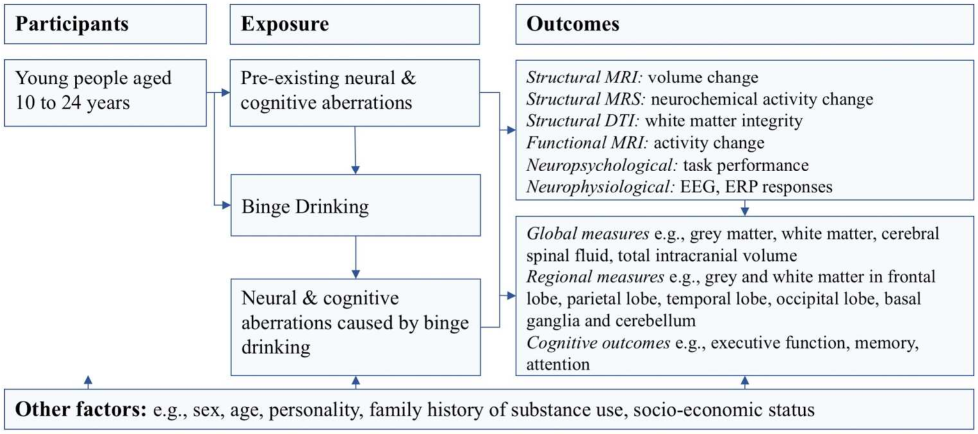

Logic model: Antecedents and consequences of binge drinking in adolescents and young adulthood.

199x91mm (300 x 300 DPI)

Supplementary File

Supplement Table 1: PRISMA-P (Preferred Reporting Items for Systematic review and Meta-Analysis Protocols) 2015 checklist: recommended items to address in a systematic review protocol* [1]

Section and topic	Item No	Checklist item	Information reported
**ADMINISTRATIVE INFORMATION**			
Title:			
Identification	1a	Identify the report as a protocol of a systematic review	☒
Update	1b	If the protocol is for an update of a previous systematic review, identify as such	☐
Registration	2	If registered, provide the name of the registry (such as PROSPERO) and registration number	☒
Authors:			
Contact	3a	Provide name, institutional affiliation, e-mail address of all protocol authors; provide physical mailing address of corresponding author	☒
Contributions	3b	Describe contributions of protocol authors and identify the guarantor of the review	☒
Amendments	4	If the protocol represents an amendment of a previously completed or published protocol, identify as such and list changes; otherwise, state plan for documenting important protocol amendments	☐
Support:			
Sources	5a	Indicate sources of financial or other support for the review	☒
Sponsor	5b	Provide name for the review funder and/or sponsor	☐
Role of sponsor or funder	5c	Describe roles of funder(s), sponsor(s), and/or institution(s), if any, in developing the protocol	☐
**INTRODUCTION**			
Rationale	6	Describe the rationale for the review in the context of what is already known	☒
Objectives	7	Provide an explicit statement of the question(s) the review will address with reference to participants, interventions, comparators, outcomes and study characteristics (PICOS)	☒
**METHODS**			
Eligibility criteria	8	Specify the study characteristics (such as PICOS, study design, setting, time frame) and report characteristics (such as years considered, language, publication status) to be used as criteria for eligibility for the review	☒
Information sources	9	Describe all intended information sources (such as electronic databases, contact with study authors, trial registers or other grey literature sources) with planned dates of coverage	☒
Search strategy	10	Present draft of search strategy to be used for at least one electronic database, including planned limits, such that it could be repeated	☒
Study records:			
Data management	11a	Describe the mechanism(s) that will be used to manage records and data throughout the review	☒
Selection process	11b	State the process that will be used for selecting studies (such as two independent reviewers) through each phase of the review (that is, screening, eligibility and inclusion in meta-analysis)	☒
Data collection process	11c	Describe planned method of extracting data from reports (such as piloting forms, done independently, in duplicate), any processes for obtaining and confirming data from investigators	☒

For peer review only

Data items	12	List and define all variables for which data will be sought (such as PICOS items, funding sources), any pre-planned data assumptions and simplifications	☒
Outcomes and prioritization	13	List and define all outcomes for which data will be sought, including prioritization of main and additional outcomes, with rationale	☒
Risk of bias in individual studies	14	Describe anticipated methods for assessing risk of bias of individual studies, including whether this will be done at the outcome or study level, or both; state how this information will be used in data synthesis	☒
Data synthesis	15a	Describe criteria under which study data will be quantitatively synthesised	☒
	15b	If data are appropriate for quantitative synthesis, describe planned summary measures, methods of handling data and methods of combining data from studies, including any planned exploration of consistency (such as I^2, Kendall's τ)	☒
	15c	Describe any proposed additional analyses (such as sensitivity or subgroup analyses, meta-regression)	☐
	15d	If quantitative synthesis is not appropriate, describe the type of summary planned	☒
Meta-bias(es)	16	Specify any planned assessment of meta-bias(es) (such as publication bias across studies, selective reporting within studies)	☐
Confidence in cumulative evidence	17	Describe how the strength of the body of evidence will be assessed (such as GRADE)	☒

*** It is strongly recommended that this checklist be read in conjunction with the PRISMA-P Explanation and Elaboration (cite when available) for important clarification on the items. Amendments to a review protocol should be tracked and dated. The copyright for PRISMA-P (including checklist) is held by the PRISMA-P Group and is distributed under a Creative Commons Attribution Licence 4.0.**

For peer review only

Supplement Table 2: Search strategy for Medline

Search	Terms
1.	(alcohol OR binge OR binge drink* OR ethanol).mp
2.	(neuroimage* OR brain OR brain imag* OR magnetic resonance OR functional magnetic resonance OR diffusion tensor imag* OR MRI OR fMRI OR DTI OR MRS OR neuropsycholog* OR neurophysiolog* OR electrophysiolog* OR EEG OR ERP OR cogniti* OR verbal working memory OR episodic memory OR visuospatial working memory OR verbal fluency OR executive function OR digit symbol substitution OR reaction time OR attention).mp.
3.	(adol* OR youth OR emerging adult OR young adult OR teen*).mp.
5.	Limit 1-3 to human
6.	Limit 1-4 to English language
7.	Limit 1-5 to "Article" [Publication Type]

[mp=title, abstract, original title, name of substance word, subject heading word, keyword heading word, protocol supplementary concept word, rare disease supplementary concept word, unique identifier, synonyms].

Supplementary Reference:

1. Shamseer L, Moher D, Clarke M, Ghersi D, Liberati A, Petticrew M, et al. Preferred reporting items for systematic review and meta-analysis protocols (PRISMA-P) 2015: Elaboration and explanation. BMJ. 2015;349:g7647.

PRISMA-P (Preferred Reporting Items for Systematic review and Meta-Analysis Protocols) 2015 checklist: recommended items to address in a systematic review protocol*

Section and topic	Item No	Checklist item	Information reported
**ADMINISTRATIVE INFORMATION**			
Title:			
Identification	1a	Identify the report as a protocol of a systematic review	☒
Update	1b	If the protocol is for an update of a previous systematic review, identify as such	☐
Registration	2	If registered, provide the name of the registry (such as PROSPERO) and registration number	☒
Authors:			
Contact	3a	Provide name, institutional affiliation, e-mail address of all protocol authors; provide physical mailing address of corresponding author	☒
Contributions	3b	Describe contributions of protocol authors and identify the guarantor of the review	☒
Amendments	4	If the protocol represents an amendment of a previously completed or published protocol, identify as such and list changes; otherwise, state plan for documenting important protocol amendments	☐
Support:			
Sources	5a	Indicate sources of financial or other support for the review	☒
Sponsor	5b	Provide name for the review funder and/or sponsor	☐
Role of sponsor or funder	5c	Describe roles of funder(s), sponsor(s), and/or institution(s), if any, in developing the protocol	☐
**INTRODUCTION**			
Rationale	6	Describe the rationale for the review in the context of what is already known	☒
Objectives	7	Provide an explicit statement of the question(s) the review will address with reference to participants, interventions, comparators, outcomes and study characteristics (PICOS)	☒
**METHODS**			
Eligibility criteria	8	Specify the study characteristics (such as PICOS, study design, setting, time frame) and report characteristics (such as years considered, language, publication status) to be used as criteria for eligibility for the review	☒
Information sources	9	Describe all intended information sources (such as electronic databases, contact with study authors, trial registers or other grey literature sources) with planned dates of coverage	☒
Search strategy	10	Present draft of search strategy to be used for at least one electronic database, including planned limits, such that it could be repeated	☒
Study records:			
Data management	11a	Describe the mechanism(s) that will be used to manage records and data throughout the review	☒
Selection process	11b	State the process that will be used for selecting studies (such as two independent reviewers) through each phase of the review (that is, screening, eligibility and inclusion in meta-analysis)	☒
Data collection process	11c	Describe planned method of extracting data from reports (such as piloting forms, done independently, in duplicate), any processes for obtaining and confirming data from investigators	☒
Data items	12	List and define all variables for which data will be sought (such as PICOS items, funding sources), any pre-planned data assumptions and simplifications	☒
Outcomes and	13	List and define all outcomes for which data will be sought, including	☒

For peer review only

prioritization		prioritization of main and additional outcomes, with rationale	
Risk of bias in individual studies	14	Describe anticipated methods for assessing risk of bias of individual studies, including whether this will be done at the outcome or study level, or both; state how this information will be used in data synthesis	☒
Data synthesis	15a	Describe criteria under which study data will be quantitatively synthesised	☒
	15b	If data are appropriate for quantitative synthesis, describe planned summary measures, methods of handling data and methods of combining data from studies, including any planned exploration of consistency (such as I^2, Kendall's τ)	☒
	15c	Describe any proposed additional analyses (such as sensitivity or subgroup analyses, meta-regression)	☐
	15d	If quantitative synthesis is not appropriate, describe the type of summary planned	☒
Meta-bias(es)	16	Specify any planned assessment of meta-bias(es) (such as publication bias across studies, selective reporting within studies)	☐
Confidence in cumulative evidence	17	Describe how the strength of the body of evidence will be assessed (such as GRADE)	☒

*** It is strongly recommended that this checklist be read in conjunction with the PRISMA-P Explanation and Elaboration (cite when available) for important clarification on the items. Amendments to a review protocol should be tracked and dated. The copyright for PRISMA-P (including checklist) is held by the PRISMA-P Group and is distributed under a Creative Commons Attribution Licence 4.0.**

For peer review only
